# MKT-GMM: A Motion Knowledge Transferring Framework for Robot Trajectory Adaptation to Variable Via-Points

**DOI:** 10.3390/biomimetics11050351

**Published:** 2026-05-19

**Authors:** Congcong Ye, Chengxing Wu, Miao Luo, Lunping Li, Xu Tang

**Affiliations:** 1 Huazhong Institute of Electro-Optics, Wuhan 430223, China; yecong_mse@hust.edu.cn (C.Y.); luomiaocq@foxmail.com (M.L.); 13554541717@139.com (L.L.); 2 State Key Laboratory of Intelligent Manufacturing Equipment and Technology, School of Mechanical Science and Engineering, Huazhong University of Science and Technology, Wuhan 430074, China; tangx@hust.edu.cn

**Keywords:** learning from demonstration, GMM/GMR, motion knowledge representation, kinesthetic teaching, trajectory optimization

## Abstract

Human motion provides a valuable source of information for robotic skill acquisition, and Learning from Demonstration (LfD) has been widely adopted as an intuitive paradigm for enabling robots to learn tasks from human demonstrations. However, the lack of an explicit representation of transferable motion knowledge significantly limits the adaptability of LfD when tasks involve varying spatial constraints or environmental configurations. To address this challenge, this paper proposes a motion representation framework based on two fundamental properties of motion and introduces a novel Motion Knowledge Transferring Gaussian Mixture Model (MKT-GMM) for trajectory generalization across related tasks. In the proposed framework, demonstration trajectories from a source task are first collected through kinesthetic teaching and encoded using a Gaussian Mixture Model (GMM), where each Gaussian component represents a local motion primitive. Transferable motion knowledge is captured by jointly preserving the statistical characteristics of individual motion primitives and the geometric relationships between adjacent primitives. For a target task in which only task constraints are specified, the learned motion knowledge is transferred by adapting the GMM parameters through affine transformations combined with constraint-error minimization, enabling feasible trajectories to be generated without additional demonstrations or model retraining. The final motions are reconstructed using Gaussian Mixture Regression (GMR), ensuring smooth and consistent trajectory generation. To further improve the robustness of trajectory transfer, a pseudo via-point mechanism is introduced to automatically generate intermediate constraints when explicit via-points are unavailable. Experiments conducted on a robotic manipulation platform, including handwriting motion learning and pick-and-place tasks under varying task configurations, demonstrate that the proposed method effectively captures transferable motion knowledge and achieves reliable trajectory generalization for previously unseen tasks.

## 1. Introduction

With the rapid advancement of industrial automation, robot programming has become increasingly critical across diverse domains, including manufacturing, healthcare, agriculture, and smart home applications [1,2]. As robots are expected to operate in increasingly complex and dynamic environments, efficient and flexible programming paradigms have become essential for enabling the rapid deployment of robotic systems. Substantial progress has been achieved in programming methodologies, such as online programming [3], Offline Programming (OLP) [4], Learning from Demonstration (LfD) [5], and Virtual Reality (VR)-based programming [6]. These approaches provide different levels of flexibility for task specification, ranging from traditional code-based programming to more intuitive human–robot interaction interfaces. Despite these advances, most approaches still heavily rely on expert knowledge or large-scale datasets, limiting their scalability and hindering the wider adoption of robotic automation [7,8]. For example, traditional programming methods require extensive domain expertise, while data-driven learning approaches often demand a large number of demonstrations or training samples. Therefore, there is a growing need for intuitive and efficient programming paradigms that can reduce the complexity of task instruction and enable the seamless transfer of human expertise to robotic execution. In particular, enabling robots to learn motion skills from a small number of human demonstrations and generalize them to new task situations remains a key challenge in robotic learning.

Learning from Demonstration (LfD), also known as Programming by Demonstration (PbD) or Imitation Learning, has emerged as one of the most effective methods for teaching robots. LfD allows a robot to acquire skills by observing and imitating a human or another robot [9], thus removing the need for specialized programming expertise and enabling non-experts to demonstrate tasks directly [10]. More importantly, the learned motion knowledge should be able to generalize across tasks with different spatial constraints or environmental configurations. To address this challenge, researchers have employed probabilistic modeling techniques, such as Gaussian Mixture Models (GMMs) combined with Gaussian Mixture Regression (GMR), to encode demonstrated trajectories and generate smooth, generalized motions [11]. These probabilistic approaches provide compact statistical representations of human motion and allow smooth trajectory reproduction from demonstrations. However, these models are typically task-specific and may struggle to generalize to novel tasks or varying intermediate and terminal positions [12].

To overcome these limitations, various extensions of GMM/GMR have been proposed [13,14], most of which adjust only Gaussian centers to accommodate new environments [15]. For example, Hersch et al. [16] integrate dynamical system control with statistical learning to handle variations in initial conditions and environmental perturbations, while Calinon et al. [17] optimize imitation metrics to generalize task skills across contexts. Other methods, such as those of Xiao et al. [18] and Feng et al. [19], incorporate force control strategies or enhanced optimization techniques to improve trajectory smoothness and learning accuracy from limited demonstrations. Task-Parameterized GMM (TP-GMM) [20] further extends GMMs to modulate both means and covariances according to task frames. Nevertheless, TP-GMM relies on human-defined frames, which may be redundant or omit critical task constraints [21]. Kernelized Movement Primitives (KMPs) [22] and motion segmentation approaches [23] offer alternatives for adapting skills, but often lack explicit probabilistic representation or require manual design of conditional distributions [24]. Although these approaches improve the adaptability of learned trajectories, most of them focus on trajectory adaptation rather than explicitly representing transferable motion knowledge across tasks.

Moreover, in real-world demonstrations, human teachers often focus on immediate task completion without fully exploiting robotic capabilities, while task constraints and environmental uncertainties are complex or unknown [25]. Consequently, demonstrations collected from a source task may not be directly applicable to related tasks with different spatial configurations or constraint conditions. These factors exacerbate difficulties in skill transfer. Therefore, developing motion representations that capture inherent relationships among task constraints becomes crucial for enabling effective knowledge transfer across tasks. Capturing inherent relationships among variable constraints across tasks through parametric modeling can enable the effective transfer of motion knowledge from one task to related tasks.

Motivated by these challenges, this paper proposes a motion knowledge transference method to generalize learned trajectories from a source task to related target tasks under varying task constraints. The proposed approach integrates Learning from Demonstration (LfD) with a motion knowledge transference mechanism to capture transferable motion characteristics embedded in human demonstrations. In this framework, human motions are represented as a sequence of elementary actions encoded by Gaussian Mixture Models (GMMs), where each Gaussian component corresponds to a local motion primitive [26]. To facilitate knowledge transfer across tasks, both the statistical characteristics of individual motion primitives and the geometric relationships between adjacent primitives are preserved, thereby maintaining the structural consistency of the learned motion representation. Based on this representation, motion knowledge obtained from a source task is adapted to new task configurations through a GMM/GMR-based motion planning process combined with task-constraint adaptation. The effectiveness of the proposed method is validated through experiments conducted on a robotic manipulation platform, including handwriting motion learning and pick-and-place tasks under varying task configurations. The main contributions of this study are as follows:

(1) A Motion Knowledge Transferring Gaussian Mixture Model (MKT-GMM) framework is proposed to enable trajectory generalization across related tasks. In the proposed framework, transferable motion knowledge is represented by jointly preserving the statistical characteristics of elementary actions and the geometric relationships among adjacent actions, allowing effective skill transfer without requiring additional demonstrations or model retraining.

(2) An integrated parameter adaptation strategy is developed by combining affine transformation estimation with task-constraint error minimization. Through this strategy, the parameters of the learned Gaussian mixture model can be systematically adjusted according to new task constraints, ensuring that the generated trajectories remain smooth while satisfying task-specific requirements.

(3) A pseudo-via-point mechanism is introduced to extend the applicability of the proposed framework to both simple and complex motion structures. By automatically generating intermediate constraints when explicit via-points are unavailable, the proposed method provides a unified parameter adaptation scheme that improves the robustness of trajectory transfer across diverse motion categories.

The rest of this paper is organized as follows: In Section 2, the motivation of considering an adaptive model is presented to interpret the human motion in LfD. The overall framework for the motion knowledge transference is presented in Section 2. Section 3 concentrates on the recognition of the source tasks, the motion knowledge representation and how to transfer the previously-explored human motion knowledge representation to the target task. In Section 4, the experimental validation of the method is performed with several experiments. Finally, the conclusion and future work are presented in Section 5.

## 2. Motivation

LfD has been widely recognized as an effective approach to improving the practicality and usability of robotic systems across diverse applications [27]. A key requirement of LfD is the ability to generalize knowledge from prior demonstrations and to transfer skills seamlessly to new tasks. In other words, if a human operator can perform a task, the learned knowledge should allow the robot to replicate the demonstrated motion and adapt it to accomplish the target task [28].

Achieving such generalization requires a high degree of flexibility in robot programming. Pre-programmed routines alone are often insufficient to handle tasks with varying constraints, as robots must be capable of adapting to different environments and unforeseen situations [1,29]. LfD provides this essential adaptability by encoding human demonstrations or explicit instructions into structured motion knowledge representations. These representations then serve as the basis for generating appropriate robot actions in the target task environment. The concrete problem of this paper is to adapt the GMM model learned from source demonstrations to new via-point constraints without re-demonstration.

**Figure 1:**
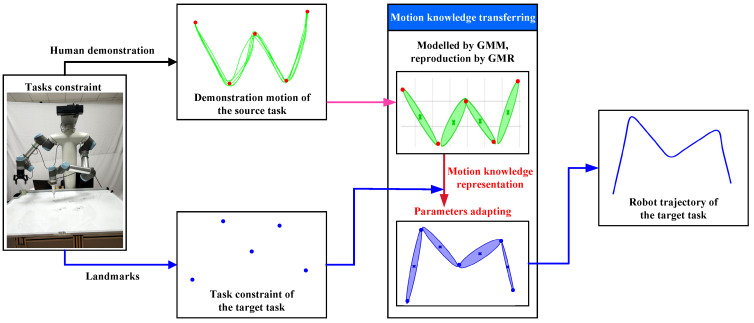
Robot programming by motion knowledge representation transferring for different constraint tasks.

Crucially, the effectiveness of this approach depends on how human motion is interpreted and structured. In LfD, human motion is commonly perceived as a sequence of elementary actions, or action primitives [30,31]. To facilitate generalization, these primitives should be extracted and refined from demonstration data. As illustrated in Figure 1, human motion is often constrained by task-specific factors, including start points, intermediate via-points, and end points [32]. The most informative cues for generalizing motion are the transitions between successive primitives [33], while the relationships among task parameters allow each primitive to be appropriately scaled dilated, compressed, or rotated according to task requirements [23]. By focusing on structured motion knowledge representation, robots can leverage this information to infer task constraints accurately and generate suitable trajectories for new tasks.

In this section, some notations and definitions used in this paper are introduced as follows:

- T denotes the task of the robot, $T^{src}$ and $T^{tar}$ refer to the source task and target task, respectively.
- C denotes the task constraints, namely the locations of intermediary via-points. $C^{src}$ and $C^{tar}$ refer to the task constraints of $T^{src}$ and $T^{tar}$, respectively.
- Θ denotes one Gaussian Mixture Model, and its parameters are mainly composed of the prior probability π, mean vector μ, and covariance matrix Σ.

### 2.1. Problem Formulation

Assume that a robotic system has acquired prior human motion knowledge from a source task $T^{src}$, on which it has previously applied distinct exploratory motion knowledge representations corresponding to different task constraints $C^{src}$. These motions are typically composed of feature observations captured by the observation models from $T^{src}$. When the robot is tasked with learning the motion of a target task $T^{tar}$, the shared properties between $T^{src}$ and $T^{tar}$ can be leveraged to improve learning efficiency. Following this rationale, the procedure is defined as follows:

- A set of *M* via-points is designed to encode the task constraints $C^{src}={\left\{p_{m}^{src}\right\}}_{m=1}^{M}$ and $C^{tar}={\left\{p_{m}^{tar}\right\}}_{m=1}^{M}$ for $T^{src}$ and $T^{tar}$, respectively.
- The Gaussian Mixture Model (GMM) parameters for the source task are defined as $\Theta^{src}={\left\{\pi_{k}^{src},\mu_{k}^{src},\Sigma_{k}^{src}\right\}}_{k=1}^{K}$, derived from the demonstration dataset $\xi^{src}=\left\{\xi_{t}^{src},\xi_{s}^{src}\right\}$, where *t* and *s* denote temporal and spatial variables, respectively. K is manually set only for controlled experiments, and can be automatically determined by BIC or AIC criteria in practical applications.
- The robot maintains a prior motion knowledge representation Π associated with $T^{src}$.

**Figure 2:**
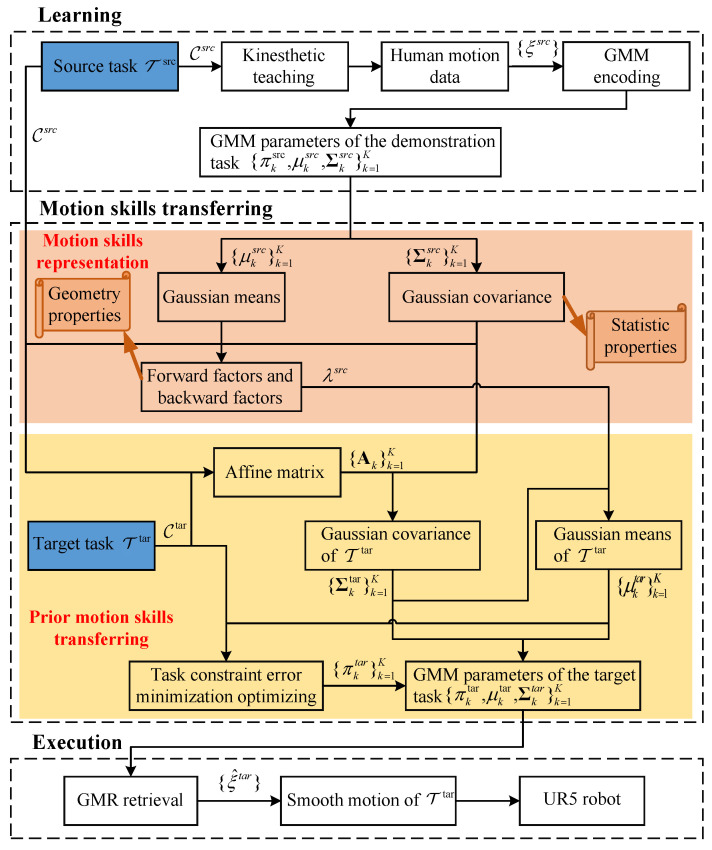
Information flow across the complete motion knowledge transferring system based on GMM/GMR method [26]. The human motion knowledge representation is delineated as two desirable common properties for $T^{src}$ and $T^{tar}$: statistic properties and geometry properties. The GMM parameters of $T^{tar}$ are estimated in the process of active prior human motion knowledge transferring. GMR is used to retrieve the motion of $T^{tar}$, and finally, the UR5 is driven to complete $T^{tar}$.

Within the MKT-GMM framework, the robot actively constructs reliable models according to the mapping $\left\{\Theta^{src},C^{src},C^{tar}\right\}\overset{\Pi }{⟶}\Theta^{tar}$, thereby generating smooth generalized trajectories ${\hat{\xi }}^{tar}=\left\{\xi_{t}^{tar},{\hat{\xi }}_{s}^{tar}\right\}$ for $T^{tar}$. Observations for both $C^{src}$ and $C^{tar}$ can be obtained in advance. The MKT-GMM framework comprises three primary phases: learning, motion knowledge transferring, and execution. An overview of the framework is presented in Figure 2. In this framework, human motion knowledge is represented through two essential shared properties for $T^{src}$ and $T^{tar}$: statistical properties and geometric properties. The GMM parameters for $T^{tar}$ are estimated through the process of active prior motion knowledge transfer, after which the UR5 manipulator executes the target task.

During the learning phase, human demonstrators perform the source task $T^{src}$ according to the specified task constraints, while the robot end-effector trajectory is recorded as a function of time. The demonstrations are subsequently temporally aligned, and the demonstration data are encoded into a GMM. In the motion knowledge transferring phase, the robot extracts the essential task correlations between source and target tasks. Each Gaussian component is interpreted as a motion primitive, and the geometric relationships between adjacent components are regarded as critical motion knowledge. Additionally, a transition matrix $A_{k}$ is derived based on $C^{src}$ and $C^{tar}$. Finally, the GMM parameters for the target task $T^{tar}$ are estimated using $C^{tar}$, the motion primitives, the geometric properties, and the transition matrix $A_{k}$. In the execution phase, the robot generates smooth, generalized trajectories for $T^{tar}$ through Gaussian Mixture Regression (GMR). This process leverages the automatically learned temporal dependencies from demonstrations, while reusing prior motion knowledge to guide trajectory planning.

### 2.2. What to Imitate and Transfer

When a human demonstrates an exploratory action to a robotic system, multiple feature observations are perceived. The prior motion knowledge is established based on the source task constraint $C^{src}$ and the corresponding Gaussian Mixture Model (GMM) of $T^{src}$. To learn the GMM for the target task $T^{tar}$, two fundamental properties are delineated to be preserved between $T^{src}$ and $T^{tar}$: (1) preservation of the statistical properties of elementary actions, and (2) preservation of the geometric relationships among adjacent elementary actions.

(1) Objective 1—Statistical Properties: Human actions are typically perceived as a sequence of distinct elementary actions. Previous research [26] has demonstrated that each Gaussian component in the GMM/GMR framework can be directly regarded as an elementary action. For the corresponding Gaussian components of $T^{src}$ and $T^{tar}$, an affine matrix $A_{k}$ is defined to project each covariance matrix $\Sigma_{k}^{src}$ to $\Sigma_{k}^{tar}$ while preserving the correlation structure. This ensures that the statistical characteristics of individual elementary actions are maintained during knowledge transfer.

(2) Objective 2—Geometric Properties: To reproduce anthropomorphic motion from human demonstrations, the GMM of $T^{tar}$, denoted as $\Theta^{tar}$, must preserve the geometric relationships between adjacent Gaussian components. For the source task $T^{src}$, $\lambda ={\left\{\lambda_{k}^{f},\lambda_{k}^{b}\right\}}_{k=1}^{K}$ is defined as the set of junction factors between adjacent Gaussian components. These factors encode the relative positional relationships among neighboring components, ensuring that the spatial structure of the demonstrated motion is retained in the target task.

### 2.3. How to Imitation and Transfer

Based on the prior motion knowledge representation Π of $T^{src}$, the robot can learn the GMM parameters $\Theta^{tar}$ by associating its target task $C^{tar}$. As for the GMM parameters $\Theta^{tar}=$$\{\pi_{k}^{tar},$$\mu_{k}^{tar},$$\Sigma_{k}^{tar}{\}}_{k=1}^{K}$, their estimation process will be detailed in Section 3.

## 3. Motion Knowledge Representation

This section describes the proposed active prior motion knowledge transferring algorithm in detail. First, we classify the various human motion into two categories in Section 3.1. Then, we define the motion knowledge representation in Section 3.2. Furthermore, finally, in Section 3.3 and Section 3.4, we present the parameter adapting for the different target tasks of MKT-GMM.

### 3.1. Human Motion Classification

Dealing with the full spectrum of human motions represents a substantial workload, as daily life encompasses a wide variety of motion patterns. In this study, these diverse human motions are encoded within a unified GMM/GMR framework and subsequently classified into two categories. The classification criterion is based on the number of Gaussian components present between the start and goal via-points of a motion. As summarized in Table 1, handwritten letters serve as a representative example of typical human motion. According to the proposed classification, such handwriting motions can be decomposed into two cases:

- Case 1: only one Gaussian component exists between the start and goal via-points.
- Case 2: more than one Gaussian component exists between the start and goal via-points.

**Figure 3:**
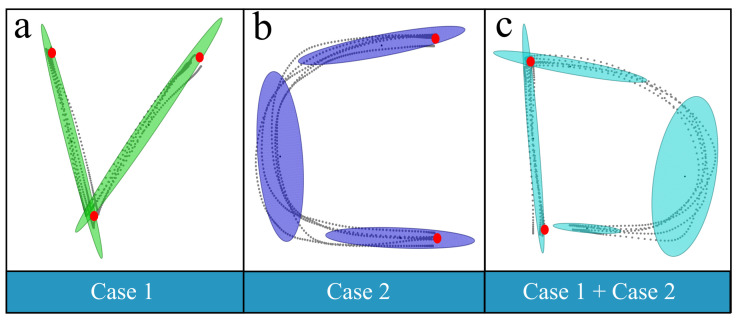
Samples (“*V*”, “*C*” and “*D*”) of two categories for human motion.

Figure 3 illustrates examples of handwritten letters (“*V*”, “*C*”, and “*D*”), where the red points denote the via-points of the handwriting motion. For the letter “*V*”, three keypoints are identified, with only a single Gaussian component connecting the start and goal via-points, corresponding to Case1. In the case of “*C*”, only the start and goal via-points are available, with three Gaussian components connecting them, corresponding to Case2. The handwriting motion of “*D*” represents a combination of Case1 and Case2, demonstrating that complex motions can comprise multiple motion types according to the classification criterion.

### 3.2. Motion Knowledge Representation of Case 1

**Figure 4:**
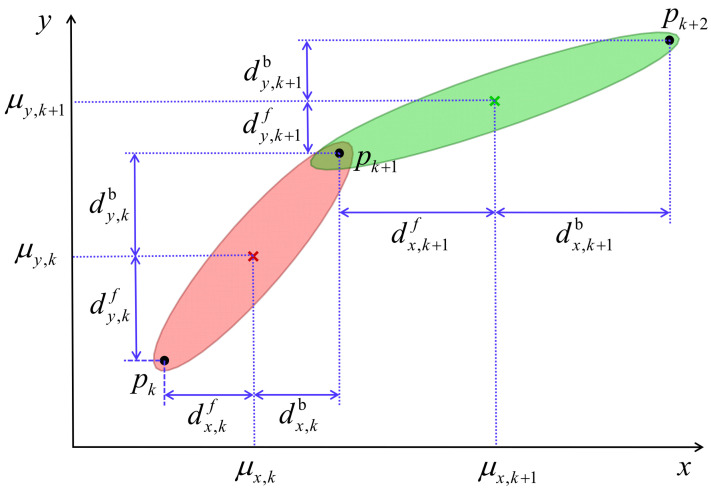
The geometry relationship of the adjacent Gaussian component *k* and k+1 of the given task T with via-points $p_{k}$, $p_{k+1}$ and $p_{k+2}$.

In this section, the motion knowledge representation is formally defined. The representation of motion skills for Case1 is discussed first. For clarity, the demonstration process is simplified in the $R^{2}$ space (e.g., assuming motion in the x − y plane, ξs=x,y). As illustrated in Figure 4, $\left\{\mu_{x,k},\mu_{y,k}\right\}$ and $\left\{\mu_{x,k+1},\mu_{y,k+1}\right\}$ denote the means of the *k*th and (k+1)th Gaussian components, respectively. The forward distance vector of the *k*th Gaussian component is defined as $d_{k}^{f}=\left\{d_{x,k}^{f},d_{y,k}^{f}\right\}$, while the backward distance vector is $d_{k}^{b}=$$\left\{d_{x,k}^{b},d_{y,k}^{b}\right\}$. The task constraint C is expressed as $p={\left\{p_{x,k},p_{y,k}\right\}}_{k=1}^{K+1}$.

The statistical representation of each Gaussian component is given by the mean and covariance pair $\mu_{s,k},\Sigma_{s,k}$, where $\mu_{k}=\left\{\mu_{t,k},\mu_{s,k}\right\}$, $\mu_{s,k}=\left\{\mu_{x,k},\mu_{y,k}\right\}$, $\Sigma_{k}=\left[\begin{array}{ccc} \Sigma_{tt,k} & \Sigma_{tx,k} & \Sigma_{ty,k}\\ \Sigma_{xt,k} & \Sigma_{xx,k} & \Sigma_{xy,k}\\ \Sigma_{yt,k} & \Sigma_{yx,k} & \Sigma_{yy,k} \end{array}\right]$, $\Sigma_{s,k}=diag\left(\left[\Sigma_{xx,k},\Sigma_{yy,k}\right]\right)$. The distance matrix for the entire trajectory is defined as $d=d_{k}^{f},d_{k}^{b}{k=1}^{K}$, where $d^{f}k$ and $d_{k}^{b}$ are computed as detailed below.

(1) $$\begin{array}{c} \left.\begin{array}{c} d_{k}^{f}\;=\;\mu_{s,k}-p_{k} \end{array}\right. \end{array}$$

(2) $$\begin{array}{c} \left.\begin{array}{c} d_{k}^{b}\;=p_{k+1}-\mu_{k} \end{array}\right. \end{array}$$

**Definition** **1** (Geometric Factors)**.** *The relative positional relationship between adjacent Gaussian components plays a critical role in GMM-based motion representation, as it directly determines the transfer trajectory connecting successive components. To quantify this relationship, a set of geometric factors geometries factors $\lambda ={\left\{\lambda_{k}^{f},\lambda_{k}^{b}\right\}}_{k=1}^{K}$ is introduced, where $\lambda^{f}k$ and $\lambda_{k}^{b}$ correspond to the forward and backward factors, respectively. These factors are computed according to Equations *(3) *and *(4)*, providing a rigorous basis for evaluating inter-component spatial relationships and facilitating accurate motion knowledge transfer.*

(3) $$\begin{array}{c} \left.\begin{array}{cc} \lambda_{k}^{f}\; & =\;\frac{d_{k}^{f}}{\sqrt{3\Sigma_{s,k}}} \end{array}\right. \end{array}$$

(4) $$\begin{array}{c} \left.\begin{array}{cc} \lambda_{k}^{b}\; & =\;\frac{d_{k}^{b}}{\sqrt{3\Sigma_{s,k}}} \end{array}\right. \end{array}$$

*In summary, the definitions of the Gaussian component statistics, distance matrices, and geometric factors collectively establish a structured motion knowledge representation. This representation captures both the probabilistic and geometric properties of demonstrated trajectories, forming the foundation for subsequent parameter adaptation and trajectory generation methods, which will be detailed in the following sections.*

### 3.3. Parameter Adapting of Case 1 via MKT-GMM

**Figure 5:**
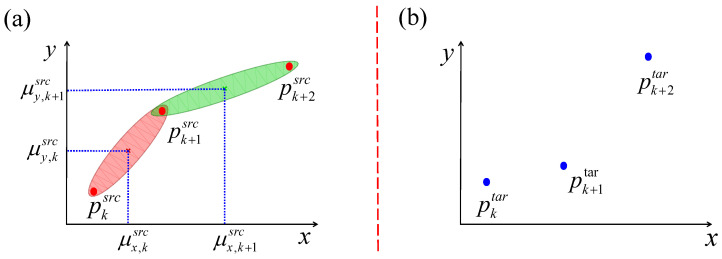
A sample of task generalization of Case1. (**a**) the source task $T^{src}$ which has been encoded by GMM, and (**b**) the task constraint of target task $T^{task}$.

A representative example of task generalization is illustrated in Figure 5. As shown in Figure 5a, the source task $T^{src}$ is encoded using the GMM method, where ${\left\{p_{x,k}^{src},p_{y,k}^{src}\right\}}_{k=1}^{K+1}$ represent the corresponding via-points. Figure 5b depicts the target task $T^{tar}$, for which only the via-points ${\left\{p_{x,k}^{tar},p_{y,k}^{tar}\right\}}_{k=1}^{K+1}$ are available. The objective is to transfer the motion skills learned from $T^{src}$ to $T^{tar}$ and subsequently construct the model for $T^{tar}$.

**Definition** **2** (Affine matrix)**.** *For each corresponding Gaussian component in $T^{src}$ and $T^{tar}$, the affine matrix $A_{k}$ is defined as a linear transformation matrix that projects $\Sigma_{k}^{src}$ to $\Sigma_{k}^{tar}$. The linear transformation factor is estimated as described in Equation *(5)*. In the specific case of robot trajectory learning, the time step ξt is considered as the input, and its distribution does not affect the output of GMR. Consequently, the linear transformation factor can be expressed as $\gamma_{t,k}=1\left(k=1:K\right)$ of $\xi_{t}$. The affine matrix $A_{k}$ for Gaussian component k is then assembled accordingly.*

(5) $$\begin{array}{cc} \gamma_{s,k} & =\frac{p_{k+1}^{tar}-p_{k}^{tar}}{p_{k+1}^{src}-p_{k}^{src}}\;,\;k=1:K-1 \end{array}$$

(6) $$\begin{array}{cc} A_{k} & =diag(\left[\gamma_{t,k},\gamma_{s,k}\right]) \end{array}$$

Based on the above definitions, human motion knowledge from $T^{src}$ can be acquired. Specifically, Definition 1 evaluates the relative positional relationships among adjacent Gaussian components, while Definition 2 enables autonomous computation of affine transformations without manual specification. Using these mechanisms, the parameters of $T^{tar}$ can be estimated by preserving the statistical and geometric properties outlined in Section 3.2. Through such prior motion knowledge transfer, $T^{tar}$ inherits the essential motion properties of $T^{src}$.

For $T^{tar}$, the parameters of the Gaussian Mixture Model $\Theta^{tar}={\left\{\pi_{k}^{tar},\mu_{k}^{tar},\Sigma_{k}^{tar}\right\}}_{k=1}^{K}$ are computed as follows. The covariance matrices ${\left\{\Sigma_{k}^{tar}\right\}}_{k=1}^{K}$ encode motion length and direction. Through the affine matrices $A_{k}$, the elementary motions of $T^{src}$ are adaptively mapped to those of $T^{tar}$, thereby preserving the *statistical properties*.

(7) $$\begin{array}{c} \Sigma_{k}^{tar}\;=\;A_{k}\Sigma_{k}^{src}A_{k}^{T} \end{array}$$

**Figure 6:**
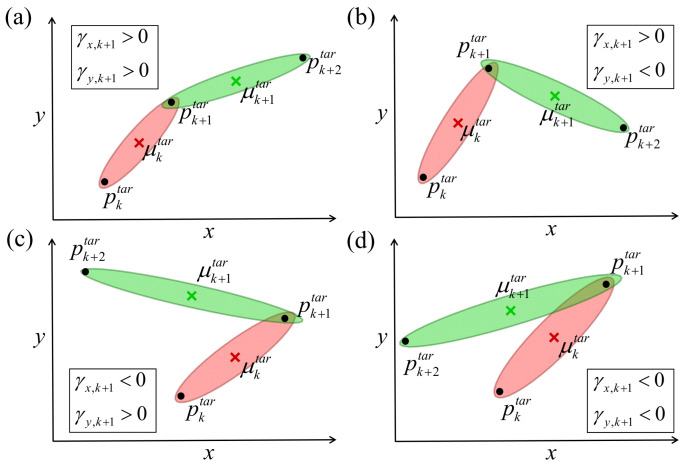
The result of Equations (8) and (9) for the different $p_{k+2}^{tar}$ with respect to Figure 5. For simplicity, only the location of via-point $p_{k+2}$ is different, i.e., $p_{k}^{tar}=p_{k}^{src}$, $p_{k+1}^{tar}=p_{k+1}^{src}$, $\mu_{k}^{tar}=\mu_{k}^{src}$, $p_{k+2}^{tar}\neq p_{k+2}^{src}$, and $\mu_{k+1}^{tar}\neq \mu_{k+1}^{src}$. With the different $p_{k+2}^{tar}$, there is a different affine matrix $A_{k}$, and the same for $\Sigma_{k+1}^{src}$ and $\mu_{k+1}^{src}$. (**a**) $\gamma_{x,k+1}>0$, $\gamma_{y,k+1}>0$, so $\kappa_{x,k+1}=1$, $\kappa_{y,k+1}=1$; (**b**) $\gamma_{x,k+1}>0$, $\gamma_{y,k+1}<0$, so $\kappa_{x,k+1}=1$, $\kappa_{y,k+1}=-1$; (**c**) $\gamma_{x,k+1}<0$, $\gamma_{y,k+1}>0$, so $\kappa_{x,k+1}=-1$, $\kappa_{y,k+1}=1$; (**d**) $\gamma_{x,k+1}<0$, $\gamma_{y,k+1}<0$, so $\kappa_{x,k+1}=-1$, $\kappa_{y,k+1}=-1$.

In addition, maintaining the *geometric properties*—in particular, the relative positions between adjacent elementary motions—is critical to ensuring similarity between $T^{src}$ and $T^{tar}$. To this end, switching consistency is imposed such that both tasks share the same forward and backward factors. The affine transformation and sign decision are constrained by global geometric factors, which reduces the sensitivity to local via-point noise. For $T^{tar}$, the signs of the scaling factors $\gamma_{x,k}$ and $\gamma_{y,k}$ significantly influence the relationship between $p_{k}$ and $\mu_{k}$, as illustrated in Figure 6. These factors determine the sign of the correlation coefficient of $\Sigma_{k}^{tar}$ in Equation (7). Accordingly, a sign(·) function is introduced in Equation (8), enabling the computation of the centers of $T^{tar}$ via Equation (9).

(8) $$\begin{array}{c} \left.\begin{array}{c} \kappa_{s,k}\;=\;sign\left(\gamma_{s,k}\right) \end{array}\right. \end{array}$$

(9) $$\left\{\begin{array}{c} \mu_{t,k}^{tar}\;=\;\mu_{t,k}^{src}\\ \mu_{s,k}^{tar}\;=\;p_{k}^{tar}+\kappa_{k}\cdot \lambda_{k}^{f}\cdot \sqrt{3\Sigma_{s,k}^{tar}} \end{array}\right.$$

Having obtained ${\left\{\Sigma^{tar}\right\}}_{k=1}^{K}$ and ${\left\{\mu^{tar}\right\}}_{k=1}^{K}$ from Equations (7) and (9), the prior probabilities ${\left\{\pi_{k}^{tar}\right\}}_{k=1}^{K}$ remain the only unknown parameters for $T^{tar}$. Since the target task lacks sufficient sample data, the expectation maximization algorithm cannot be applied directly. Instead, these parameters are estimated by formulating an optimization problem, in which ${\left\{\pi_{k}^{tar}\right\}}_{k=1}^{K}$ are treated as optimization variables. Specifically, the objective is to minimize the error between the GMR outputs and the task constraints $C^{tar}$. For each task via-point $p_{m}^{tar}$, with corresponding time step $\xi_{t,m}$, the GMR output is ${\hat{\xi }}_{\xi_{t,m}}=\left\{{\hat{\xi }}_{x,\xi_{t,m}},{\hat{\xi }}_{y,\xi_{t,m}}\right\}$. The cost function *f* and its associated constraints are defined in Equation (10), and ${\left\{\pi_{k}^{tar}\right\}}_{k=1}^{K}$ are estimated by minimizing *f*. Notably, these parameters serve as optimization weights rather than conventional prior probabilities, as their role is to minimize via-point error rather than maximize likelihood.

(10) $$\begin{array}{c} \left\{\begin{array}{c} \underset{{\left\{\pi_{k}^{tar}\right\}}_{k=1}^{K}\;}{min\;f}\;=\;\sum_{m=1}^{K+1}\left|\right|p_{m}^{tar}-{\hat{\xi }}_{\xi_{t,m}}\left|\right|\\ s.t.\begin{array}{c} \;\sum_{k=1}^{K}\pi_{k}^{tar}\;=\;1\\ \;0\;<\pi_{k}^{tar}\;<\;1\;\;k=1:K \end{array} \end{array}\right. \end{array}$$

Once the complete GMM parameters $\Theta^{tar}={\left\{\pi_{k}^{tar},\mu_{k}^{tar},\Sigma_{k}^{tar}\right\}}_{k=1}^{K}$ are determined, GMR is employed to reproduce smooth, generalized motions that preserve the essential motion properties. The primary advantage of the proposed approach lies in its provision of an analytical solution for generating reliable, transferable trajectories between similar tasks. The overall estimation process for parameter computation in Case1 is summarized in Table 2.

### 3.4. Parameters Adapting of Case2 via MKT-GMM

As discussed above, in the case of Case2 (Figure 3b), multiple Gaussian components exist between the start and goal pairs. However, due to the absence of prior via-point information between adjacent Gaussian components, it is difficult to directly implement motion knowledge representation and to calculate the corresponding affine matrices. To address this issue, a set of pseudo via-points is introduced. This strategy transforms Case2 into an equivalent form of Case1, thereby simplifying the parameter adaptation process. In this section, the handwriting motion of the letter “*C*” is taken as an illustrative example to demonstrate the introduction of pseudo-via-points and the transformation from Case2 to Case1.

**Figure 7:**
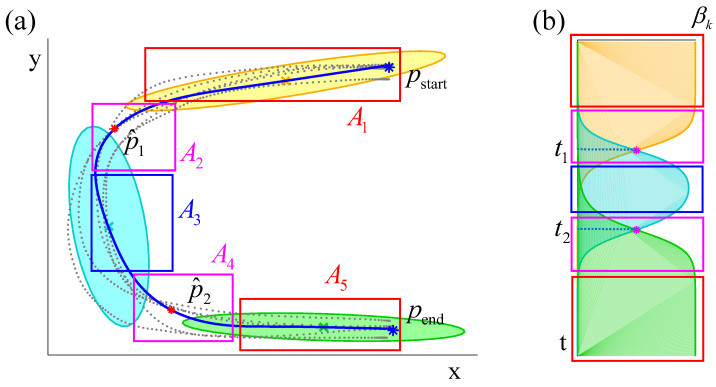
(**a**) The via-points, demonstration data, GMM and GMR of *C*. $p_{start}$ and $p_{end}$ are the task constraint, and ${\hat{p}}_{1}$ and ${\hat{p}}_{2}$ are pseudo via-points which is calculated according to $t_{1}$ and $t_{2}$ of subfigure (**b**). (**b**) The distribution of $\beta_{k}$, and $t_{1}$ and $t_{2}$ are calculated according to Equations (11) and (A1).

For each Gaussian component *k*, ${\hat{\xi }}_{s,k}$ exhibits a linear dependency with $\xi_{t}$, where $\beta_{k}$ serves as a primary contributing factor in the mixture model output ${\hat{\xi }}_{s}$. As shown in Figure 7, the motion is decomposed into five regions, $A_{1}$, $A_{2}$, $A_{3}$, $A_{4}$, and $A_{5}$, according to the distribution of $\beta_{1}$, $\beta_{2}$, and $\beta_{3}$. In $A_{1}$, the trajectory is approximately linear since $\beta_{1}\approx 1$ and $\beta_{2}\approx \beta_{3}\approx 0$. In $A_{2}$, the motion is jointly determined by $\beta_{1}$ and $\beta_{2}$ with $\beta_{3}\approx 0$. In $A_{3}$, $\beta_{2}$ is the dominant factor, while $A_{4}$ is analogous to $A_{2}$, and $A_{5}$ is analogous to $A_{1}$. Consequently, pseudo via-points are defined based on the characteristic distributions of $\beta_{k}$ across these regions.

Particular attention should be given to regions $A_{2}$ and $A_{4}$, as the motion in $A_{1}$, $A_{3}$, and $A_{5}$ can be approximated as linear. The mixture output ${\hat{\xi }}_{s}$ is expressed as ${\hat{\xi }}_{s}=\beta_{1}{\hat{\xi }}_{s,1}+\beta_{2}{\hat{\xi }}_{s,2}+\beta_{3}{\hat{\xi }}_{s,3}$. In $A_{2}$, since $\beta_{3}\approx 0$, ${\hat{\xi }}_{s}$ can be approximated as ${\hat{\xi }}_{s}=\beta_{1}{\hat{\xi }}_{s,1}+\beta_{2}{\hat{\xi }}_{s,2}$. Accordingly, the pseudo via-point ${\hat{p}}_{1}$ of $A_{2}$ is determined at the point where $\beta_{1}=\beta_{2}$. Similarly, the pseudo via-point $\hat{p}2$ of $A_{4}$ is obtained under the same condition. In general, pseudo via-points are defined at $t_{k}$ such that $\beta_{k}=\beta k+1$(k=1:K−1), as expressed in Equation (11), where the value of $t_{k}$ can be estimated following the procedure described in Appendix A.

(11) $$\begin{array}{c} \beta_{k}\left(t_{k}\right)=\beta_{k+1}\left(t_{k}\right)\;\left(k=1:K-1\right) \end{array}$$

**Figure 8:**
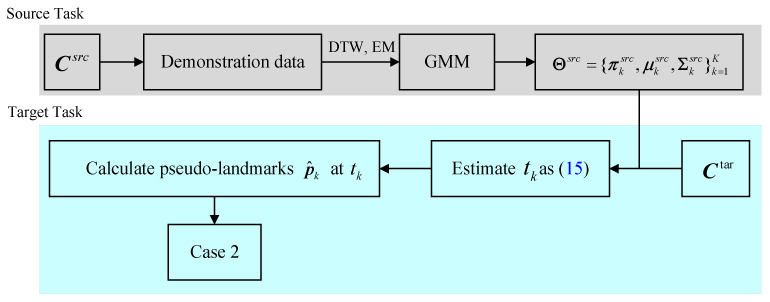
The flowchart of parameters adapting process of Case2.

The pseudo via-points ${\hat{p}}_{1}$ and $\hat{p}2$ are defined with respect to the time indices $t_{1}$ and $t_{2}$. A pseudo via-point refers to a virtual point derived from Gaussian component intersections to handle complex motions without an explicit via-point. By introducing these pseudo via-points, the scenario described in Case2 can be reformulated into the structure of Case1, thereby enabling the application of the parameter adaptation procedure illustrated in Figure 8. To facilitate this adaptation, a scale energy function $f\left(\gamma_{k,i}\right)\;\left(k=1:K,\;i=1:D\right)$ is constructed, as presented in Equations (12) and (13). This function provides a rigorous quantitative measure for evaluating parameter transformations under varying task constraints. Pseudo via-point generation is data-driven according to the Gaussian component weight $\beta_{k}$, not a heuristic strategy. It can maintain structural consistency even for irregular motion patterns.

(12) $$\begin{array}{c} f\left(\gamma_{k,i}\right)=ln\left(\frac{1}{2}\left(\gamma_{k,i}^{2}+\frac{1}{\gamma_{k,i}^{2}}\right)\right) \end{array}$$

(13) $$\begin{array}{c} H=\sum_{k=1}^{K}\sum_{i=1}^{D}f\left(\gamma_{k,i}\right) \end{array}$$

Based on this formulation, the adaptation process is designed to ensure structural consistency between the transformed and the original motion representations. In this context, the optimization problem is defined as the minimization of the proposed scale energy function, as specified in Equation (14), which guarantees that the adapted parameters preserve both the statistical and geometric properties of the underlying motion knowledge.

(14) $$\begin{array}{c} min\;H\\ s.t.\;p_{end}=p_{start}+\sum_{k=1}^{K}\kappa_{k}\left(\lambda_{k}^{f}+\lambda_{k}^{b}\right)\left(\sqrt{3\Sigma_{k}^{tar}}\right) \end{array}$$

## 4. Experimental and Results

**Figure 9:**
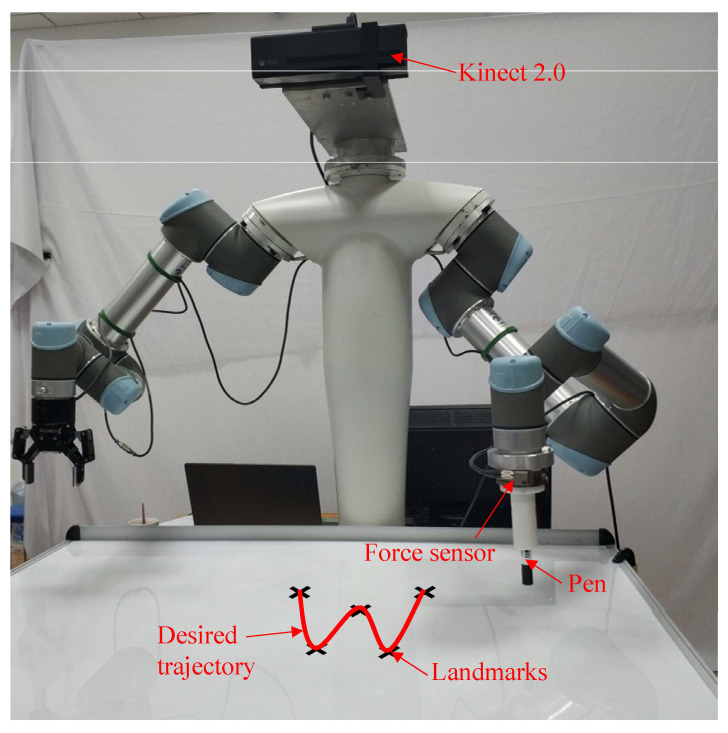
Experimental setup.

To validate the effectiveness of the proposed framework, four groups of experiments were conducted. In the first experiment, four instructors were invited to demonstrate the handwriting motion of the character “w”. The demonstrations were carried out on a UR5 robot equipped with an ATI Gamma force sensor mounted at the end-effector and a Kinect 2.0 sensor installed at the robot head, as illustrated in Figure 9. The motion skills acquired from these demonstrations were encoded using the proposed method, and different motion knowledge representations were comparatively analyzed to assess their effectiveness.

Building upon this, the second experiment examined the generalization capability of the framework in Case 1. Subsequently, the third experiment further validated the generalization performance in Case 2 by employing the handwriting motion of the character “C” as the demonstration task. Finally, to evaluate the applicability of the framework beyond handwriting tasks, a pick-and-place experiment was performed. In this scenario, the robot was required to execute the task under varying initial and target positions, thereby demonstrating the robustness of the method in handling different task configurations. Only The right robotic arm was utilized for task execution; the left arm remained stationary.

### 4.1. Experimental 1

**Figure 10:**
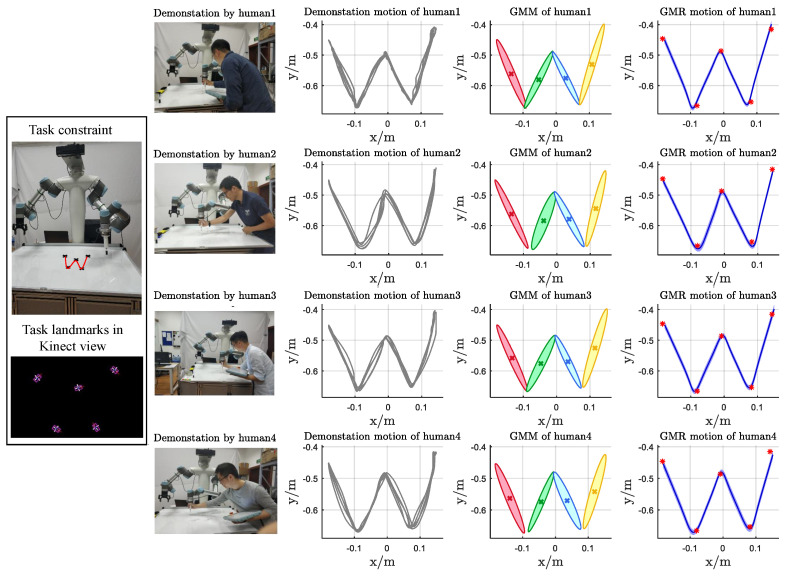
The robot was guided to accomplish the handwriting motion of the letter *w* for the same task constraint as the first column by four instructors. The demonstration dataset, GMM, and GMR for the four instructors’ motion are shown as the third column to the fifth column.

In this experiment, the motion knowledge represented by the proposed method was investigated. Four instructors were asked to demonstrate the handwriting motion of the letter “w” under the task constraint $C_{5}\left(C_{5}={\left\{p_{m}\right\}}_{m=1}^{5}\right)$. The motion data were captured using Kinect 2.0, as illustrated in the first column of Figure 10, and recorded as the nib trajectory through kinesthetic teaching. The demonstrated motions were subsequently modeled using Gaussian Mixture Models (GMMs) based on the collected data, and reproduction was performed via Gaussian Mixture Regression (GMR). In this experiment, GMMs with K=4 components were manually specified.

As shown in Figure 10, the handwriting motion datasets ${\hat{\xi }}^{human1}$, ${\hat{\xi }}^{human2}$, ${\hat{\xi }}^{human3}$, and ${\hat{\xi }}^{human4}$, corresponding to the four instructors, exhibit noticeable variations for the same task constraint $C5^{w}$. Consequently, the corresponding motion models encoded by GMM also differ. The primary differences among these models lie in their statistical and geometric properties, specifically the covariance matrices ${\left\{\Sigma_{k}\right\}}_{k=1}^{K}$ of the Gaussian components and the geometric relationship parameters λ between adjacent components, as illustrated in the third column of Figure 10. The parameter λ encodes the relative positional relationship between two consecutive Gaussian components, indicating whether or not their regions overlap. In particular, if $|\lambda_{x,k}^{f}|\leq 1$, the via-point $p_{x,k}$ is located within the Gaussian ellipsoid along the *x* dimension, and analogous conditions hold for $|\lambda_{x,k}^{b}|$, $|\lambda_{y,k}^{f}|$, and $|\lambda_{y,k}^{b}|$. Conversely, if these inequalities are not satisfied, the corresponding via-point lies outside the Gaussian ellipsoid in that dimension.

**Figure 11:**
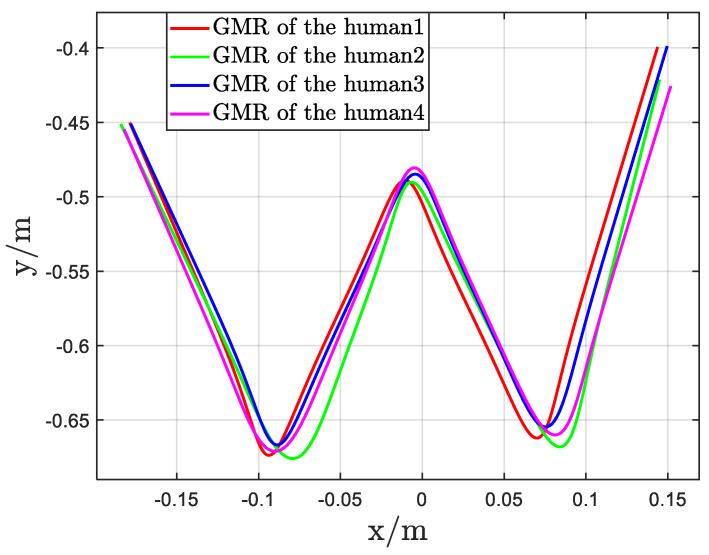
The GMR reproductio n of four instructors.

The reproduced trajectories obtained via GMR for the four instructors are presented in Figure 11, and quantitative analyses are summarized in Table 3. The correlation coefficient η and the transfer factor λ of each Gaussian component for each instructor are reported for the handwriting task. A positive value of η indicates a positive correlation between the *x* and *y* dimensions, whereas a negative value denotes a negative correlation. The transfer factor λ plays a critical role in defining the relative positional relationship between adjacent Gaussian components and directly determines the shape of the transfer curve at the corresponding via-point.

### 4.2. Experimental 2

**Figure 12:**
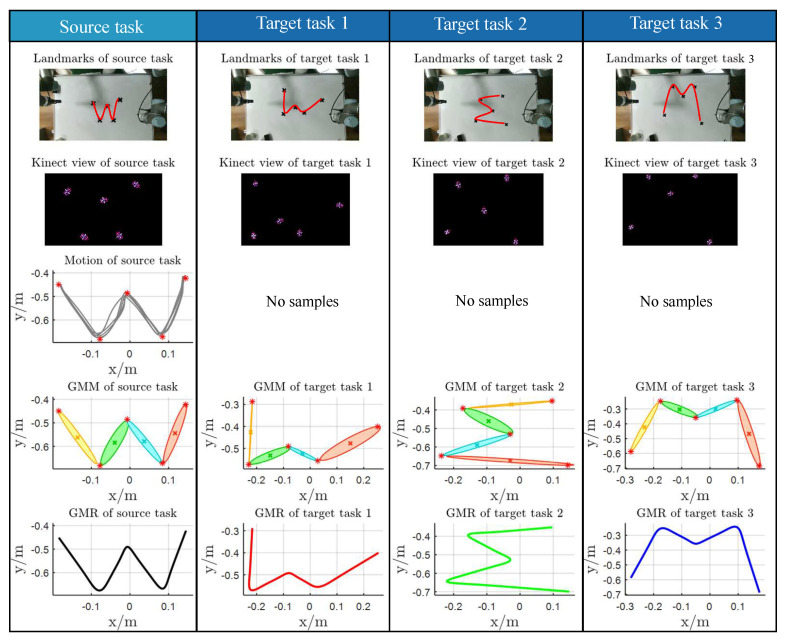
The applicability of the proposed method.

To demonstrate the generalization capability of the proposed method, the handwriting of the letter “W” was selected as the experimental task, implemented under varying task constraints, such as different positions of five via-points, using kinesthetic teaching. The task involved moving a pen, mounted on the robot end-effector, from the initial point to the endpoint while passing through intermediate points. Figure 12 presents the experimental results. The first row illustrates the task constraints for the source task and the various target tasks. Specifically, $C^{src}$ denotes the task constraint for the source task $T^{src}$, while $C^{tar1}$, $C^{tar2}$, and $C^{tar3}$ correspond to the task constraints for the different target tasks $T^{tar}$. The positions of the via-points in each task were captured using a Kinect sensor, as shown in the second column.

**Figure 13:**
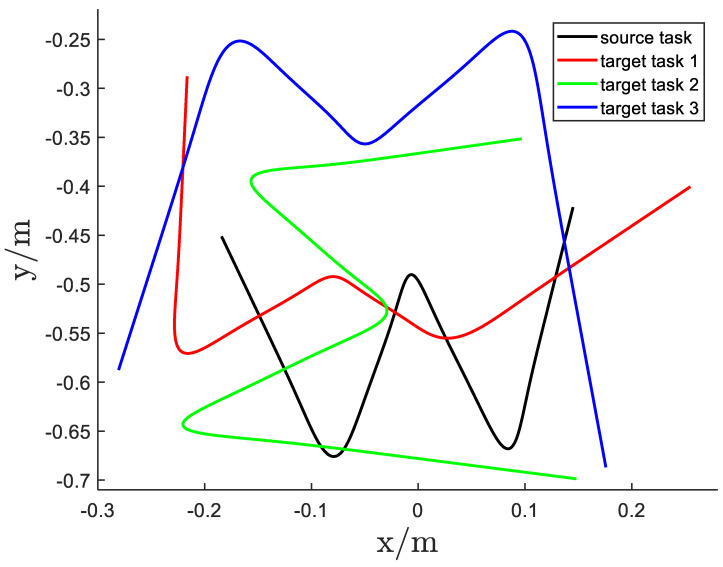
The GMR reproduction of the different tasks.

The statistical and geometric properties of the source task $T^{src}$, which were transferred to the target tasks, are summarized in Table 4, while the corresponding affine matrices $A_{k}$ for each target task relative to $T^{src}$ are presented in Table 5. In the fourth row of Figure 12, the Gaussian Mixture Models (GMMs) of the different target tasks share the same statistical and geometric properties as the source task. In the third row, only the source task provides sample data, and the task constraint error minimization procedure is employed to compute the prior probabilities ${\left\{\pi_{k}^{tar}\right\}}_{k=1}^{K}$ for the target tasks. The reproduced trajectories, shown in the fourth row, maintain consistent statistical and geometric properties during the transitions from one Gaussian component to the next. The corresponding Gaussian Mixture Regression (GMR) results for the four target tasks are presented in the fifth row, demonstrating that smooth, generalized trajectories can successfully complete each task. A comparative analysis of the reproduced trajectories across the four tasks is provided in Figure 13. ${\left\{\pi_{k}^{tar}\right\}}_{k=1}^{K}$ of the target tasks $T^{tar}$ is evaluated with Equation (10) to minimize the via-point error. At this time, the significance of ${\left\{\pi_{k}^{tar}\right\}}_{k=1}^{K}$ lies in reducing errors, rather than just the meaning of probability in GMM.

### 4.3. Experimental 3

**Figure 14:**
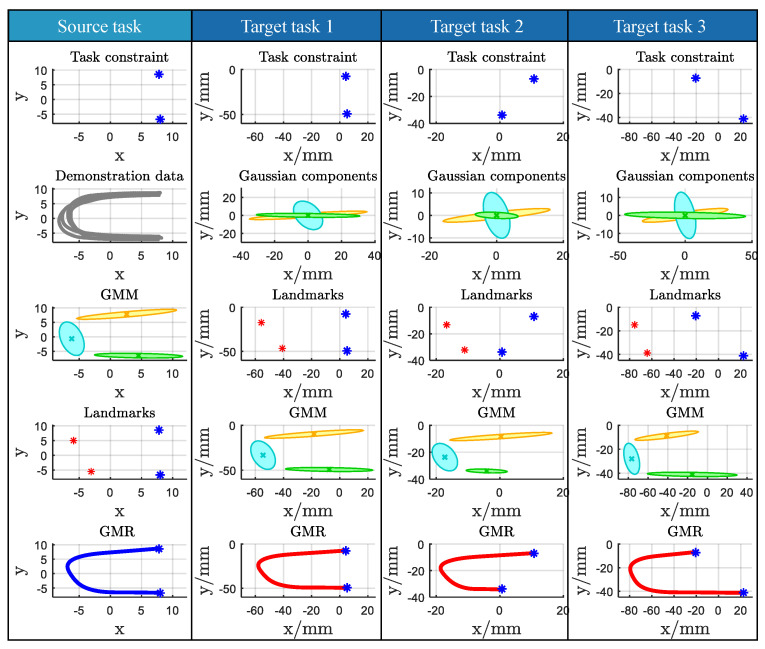
The generalization of human motion Case2 (blue star: real task via-points; red star: pseudo via-points).

In this experiment, the generalization capability of Case2 was evaluated using the LASA Handwriting Dataset library (Available in: https://gitlab.idiap.ch/rli/pbdlib-matlab, accessed on 16 March 2026), which contains 30 human handwriting motion patterns in two-dimensional space. To examine Case2, the human motion corresponding to the letter “C” was selected. The selected motion data were first encoded using K=3 (manually specified), as illustrated in the first column of Figure 14. Via-points and pseudo via-points are depicted in the fourth row, first column. For the target tasks $T^{tar1}$, $T^{tar2}$, and $T^{tar3}$, the associated task constraints are shown in the first row, columns two through four, respectively. Different task constraints correspond to different affine matrices A. Pseudo via-points and Gaussian means were sequentially computed based on the first via-point, from which the parameters of the target tasks were derived, as presented in Figure 14.

As shown in the fifth row of Figure 14, the proposed method successfully generalizes Case2. Under the given task constraints, pseudo via-points serve as key reference points, such as the connection points between adjacent Gaussian components. The generalized motions preserve the essential properties of the source task, including both statistical and geometric characteristics. This demonstrates that the proposed approach effectively transfers critical skills from the source demonstration to the robot, enabling reliable adaptation to new task conditions.

### 4.4. Experimental 4

**Figure 15:**
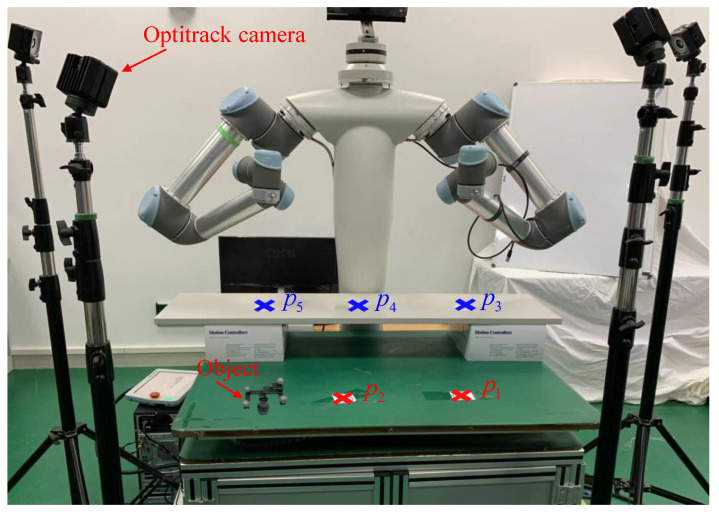
The experimental setup of experiment 4. ($p_{1}$ and $p_{2}$: pick location; $p_{3}$, $p_{4}$ and $p_{5}$: place location).

**Figure 16:**
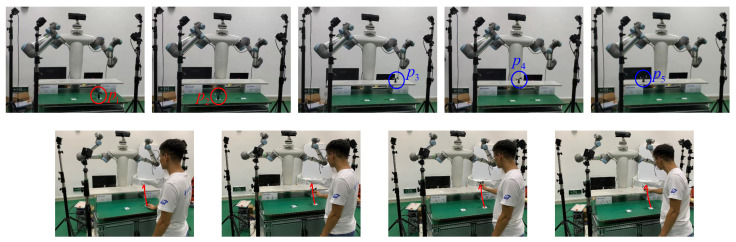
The first row displays the process of collecting the location of $p_{i}\left(i=1:5\right)$ by placing the object at the assigned via-point. The second row shows the human motion collecting process of the source task of $p_{1}\to p_{3}$.

A three-dimensional pick-and-place task was demonstrated in this experiment, involving two pick locations, $p_{1}$ and $p_{2}$, and three place locations, $p_{3}$, $p_{4}$, and $p_{5}$ (see Figure 15). The motion from $p_{1}$ to $p_{3}$ was designated as the source task, $T^{src}$, and served as the basis for transferring motion skills to other desired pick-and-place pairs, defined as target tasks, $T^{tar}$, i.e., $p_{1}\to p_{4}$, $p_{1}\to p_{5}$, $p_{2}\to p_{3}$, $p_{2}\to p_{4}$, and $p_{2}\to p_{5}$. The OptiTrack optical motion capture system was employed to record the positions of $p_{i}$(i=1:5) and to capture the human demonstration of the motion $p_{1}\to p_{3}$ (see Figure 16).

**Figure 17:**
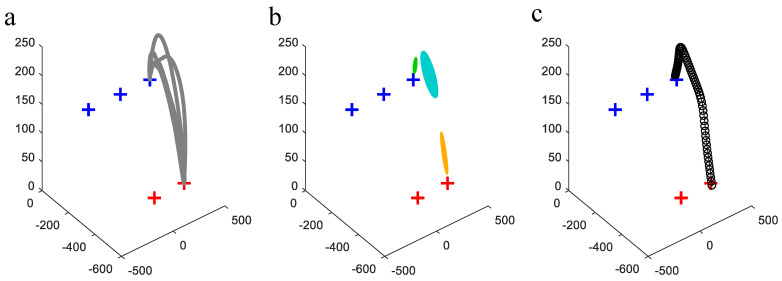
The data (**a**), GMM (**b**), and GMR (**c**) reproduction of source task motion of the pick and place pairs $p_{1}$ and $p_{3}$.

Initially, a rigid object equipped with four markers was placed at each pick and place location, $p_{i}$(i=1:5), to establish reference positions using the OptiTrack system. Subsequently, the object was moved from $p_{1}$ to $p_{3}$, and the corresponding trajectory was recorded as the source task motion. The human demonstration trajectory and its Gaussian Mixture Model (GMM) representation for $T^{src}$ are presented in Figure 17. In this experiment, GMMs with K=3 components were manually specified.

**Figure 18:**
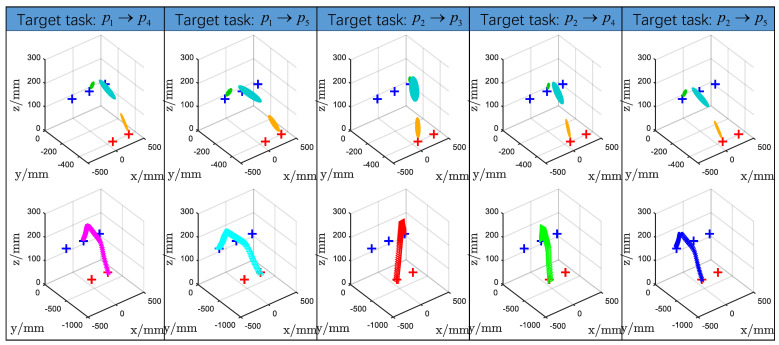
The GMM and GMR reproduction of $T^{tar}$ modeled by the proposed MKT-GMM. $T^{tar}$ share common properties with $T^{src}$.

**Figure 19:**
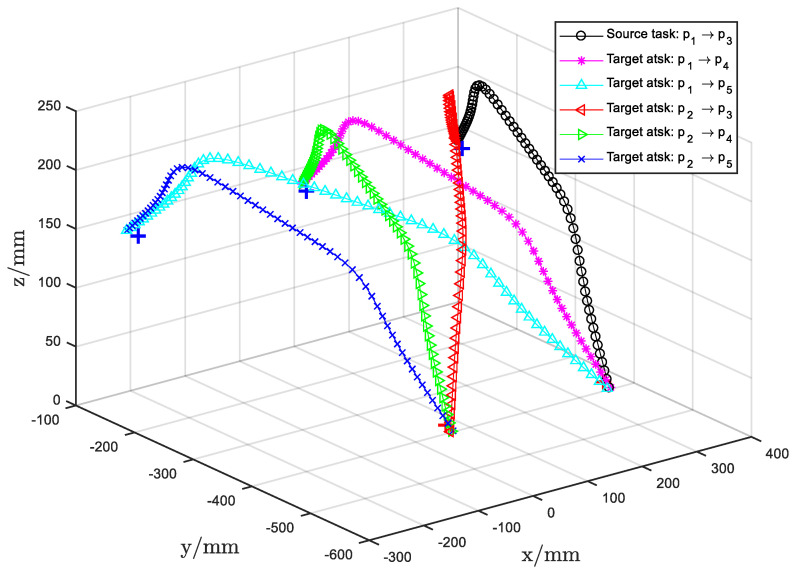
The GMR reproduc tion result of different pick and place pairs.

The GMM parameters for the target tasks, $T^{tar}$, were then estimated using the proposed motion knowledge transfer method. The resulting generalized motions for each target task are illustrated in Figure 18, while Figure 19 depicts the generalized trajectories for all evaluated pick-and-place pairs. For each specified pick-and-place pair, the motion skills encoded in $T^{src}$ were effectively represented and transferred to the corresponding $T^{tar}$. The generated target task trajectories retained the common properties of the source task, demonstrating that the proposed approach can reproduce human demonstration skills while preserving the underlying motion knowledge. This method thus enables robotic assembly tasks to be executed based on human demonstrations while maintaining the essential characteristics of the demonstrated motion.

### 4.5. Discussion

This work set out to represent and transfer human motion knowledge so that a robot can reproduce related tasks without new demonstrations. The four experiments collectively show that the proposed MKT-GMM achieves that goal by (1) extracting meaningful, task-relevant motion primitives from demonstrations, (2) adapting those primitives to new task constraints, and (3) reproducing smooth, constraint-compliant trajectories in both planar and 3D settings.

First, Experiment1 confirms that the representation captures demonstrator-specific statistical and geometric properties. GMMs fitted to different instructors encode different covariance structures and inter-component relations, and these differences are reflected in the GMR reproductions (see Figure 11 and quantitative summaries in Table 3). This shows the method effectively decomposes demonstrated behavior into elementary actions whose statistical signatures are informative for transfer.

Second, Experiment2 and 3 demonstrate two complementary adaptation mechanisms. For motions falling into Case1, simple affine scaling computed from via-point pairs suffices to adapt Gaussian covariances and means to new constraints, producing smooth target trajectories (Experiment2; illustrated in Figure 12). For Case2, where the intermediate structure is not directly constrained, the pseudo via-point strategy converts the problem into Case1 and enables similar adaptation (Experiment3; illustrated in Figure 14). In both cases the adaptation is automatic and does not require new demonstrations or manual retuning of model parameters.

Third, Experiment4 shows the approach generalizes beyond 2D handwriting to a 3D pick-and-place task. The adapted GMMs preserve the source task’s motion characteristics when transferred to new pick/place pairs, producing plausible 3D trajectories (Figure 18 and Figure 19). This indicates the framework’s potential utility in practical manipulation tasks where spatial constraints vary.

At the same time, several practical limitations and sensitivities were identified. The current pipeline is essentially open-loop: once GMM parameters for a target task are estimated, trajectory reproduction uses GMR without online correction, which limits robustness to unmodeled disturbances or perception errors. The pseudo via-point selection relies on heuristic timing and component-weight analyses; while effective for the tested examples, this heuristic may require refinement for more heterogeneous motion classes. The affine scaling and sign decisions (which affect covariance orientation and implied correlations) can also be sensitive to noisy via-point estimates. Finally, experiments used controlled optical sensing; real-world deployment will demand robustness to noisier/occluded perception.

In summary, the experiments validate that preserving both statistical properties of elementary actions and geometric relationships between adjacent actions provides a practical route for transferring demonstrated skills. The proposed MKT-GMM is an offline trajectory generalization framework, which is suitable for structured industrial scenarios with small disturbances. In future work, closed-loop visual or force feedback will be integrated to realize online trajectory correction for higher robustness. Compared with TP-GMM, MKT-GMM does not require manually defined task frames. Compared with DMP, it focuses on motion knowledge transfer across similar tasks. The proposed method will be extended to 6D pose transfer by representing orientation with quaternions.

## 5. Conclusions

This paper proposes a Motion Knowledge Transferring Gaussian Mixture Model (MKT-GMM) for robot learning from demonstration. The proposed framework preserves both the statistical characteristics of elementary actions and the geometric relationships between successive actions, enabling robust skill transfer from a source task to related target tasks. An integrated parameter adaptation framework, combining affine transformation estimation with task-constraint error minimization, is developed to generate smooth trajectories that satisfy task-specific constraints without requiring additional demonstrations. In addition, a pseudo via-point mechanism is introduced to provide a unified solution for handling both simple and complex motion structures, further improving the versatility of the proposed approach. Extensive experimental evaluations were conducted on a robotic manipulation platform through handwriting motion learning and pick-and-place tasks under varying task configurations. Comparative experiments were performed to evaluate the capability of the proposed framework in capturing demonstration variability, transferring motion knowledge across tasks, and adapting to different task constraints. In the handwriting experiments, demonstrations from multiple instructors were analyzed to examine the statistical characteristics of human motion, and the results show that the proposed representation effectively captures the variability and distinctive motion patterns among different demonstrators. In the trajectory generalization experiments, the proposed MKT-GMM was compared with direct trajectory reproduction and affine transformation–based adaptation methods, demonstrating improved accuracy in satisfying task constraints while preserving the structural consistency of the demonstrated motion. Furthermore, pick-and-place experiments under varying spatial configurations verify that the proposed method can reliably generate smooth and feasible trajectories in previously unseen task scenarios. Overall, the results indicate that the proposed framework enables effective motion knowledge transfer and achieves robust trajectory generalization under varying task constraints.

Future work will address the current limitations by developing closed-loop extensions that incorporate real-time feedback to improve accuracy and robustness. In addition, investigations will focus on cross-dimensional generalization, such as extending knowledge learned from 2D demonstrations to 3D executions, and on partial-to-complete task generalization. These directions are anticipated to broaden the applicability of the framework to increasingly diverse and complex robotic tasks, thereby advancing the state of robot learning from demonstration and contributing to the development of more autonomous and intelligent robotic systems.

## Figures and Tables

**Table 1:** Handwriting motion categories of 26 letters.

Case 1	A, E, F, H, I, K, L, M, N, T, V, W, X, Y, Z
Case 2	C, G, J, O, S, U
Case 1 + Case 2	B, D, P, Q, R

**Table 2:** Active prior motion knowledge representation and transferring process of Case1.

1. Source task demonstrations - Get $C^{src}$ of $T^{src}$ - Collect datapoint $\left\{\xi^{src}\right\}$
2. Model fitting - Set *K* (number of the Gaussian components) - *k*-means segmentation - Fit a GMM to $\xi^{src}$ with EM algorithm $\Theta^{src}={\left\{\pi_{k}^{src},\mu_{k}^{src},\Sigma_{k}^{src}\right\}}_{k=1}^{K}$
3. Motion knowledge representation - Elementary action ${\left\{\Sigma_{k}^{src}\right\}}_{k=1}^{K}$ - Geometry factors λ in Equations (3) and (4)
4. Prior motion knowledge transferring - Get $C^{tar}$ of $T^{tar}$ - Scale factors $\gamma_{k}$ in Equation (5) and affine matrix $A_{k}=\mathrm{diag}\left(\gamma_{k}\right)$ in Equation (6) - Covariance matrix ${\left\{\Sigma_{k}^{tar}\right\}}_{k=1}^{K}$ in Equation (7) - Gaussian means ${\left\{\mu_{k}^{tar}\right\}}_{k=1}^{K}$ in Equation (9) - Estimated ${\left\{\pi_{k}^{tar}\right\}}_{k=1}^{K}$ via Equation (14)
5. Reproduce of target task - Retrieve ${\hat{\xi }}^{tar}$ through GMR

**Table 3:** The comparison of the prior motion knowledge representation (η,λ) delineated by statistic properties and geometry properties of the four instructors.

	Human 1	Human 2	Human 3	Human 4
$\eta_{1}$	−0.9633	−0.9774	−0.9695	−0.9550
$\lambda_{x,1}^{f}$	1.0955	0.9819	1.1089	1.0088
$\lambda_{y,1}^{f}$	−1.0241	−1.0048	−1.0328	−1.0645
$\lambda_{x,1}^{b}$	1.2192	1.1663	1.1351	1.2990
$\lambda_{y,1}^{b}$	−0.9287	−1.0670	−0.9935	−0.9337
$\eta_{2}$	0.9690	0.8858	0.9489	0.9651
$\lambda_{x,2}^{f}$	0.6444	1.0529	0.7792	0.9372
$\lambda_{y,2}^{f}$	0.9112	1.0360	0.9824	0.9622
$\lambda_{x,2}^{b}$	1.0235	0.8886	0.9053	0.9027
$\lambda_{y,2}^{b}$	1.0073	1.0450	0.9775	0.9261
$\eta_{3}$	−0.9720	−0.9673	−0.9630	−0.9339
$\lambda_{x,3}^{f}$	0.9097	0.9807	1.0771	1.0449
$\lambda_{y,3}^{f}$	−1.0339	−1.0314	−0.9689	−0.9282
$\lambda_{x,3}^{b}$	1.2845	1.0527	1.2123	1.1055
$\lambda_{y,3}^{b}$	−0.8997	−1.0262	−0.9692	−0.9116
$\eta_{4}$	0.9598	0.9235	0.9377	0.9189
$\lambda_{x,4}^{f}$	0.6801	1.0847	0.8901	1.0663
$\lambda_{y,4}^{f}$	0.9287	1.0261	0.9991	0.9467
$\lambda_{x,4}^{b}$	0.9457	0.9079	0.7627	0.6869
$\lambda_{y,4}^{b}$	0.8703	0.9802	0.8608	1.0770

**Table 4:** The motion knowledge (η,λ) of $T^{src}$.

	k	1	2	3	4
Motion Skills					
$\eta_{k}$		−0.9774	0.8858	−0.9673	0.9235
$\lambda_{x,k}^{f}$		0.9819	1.0529	0.9807	1.0847
$\lambda_{y,k}^{f}$		−1.0048	1.0360	−1.0314	1.0261
$\lambda_{x,k}^{b}$		1.1663	0.8886	1.0527	0.9079
$\lambda_{y,k}^{b}$		−1.0670	1.0450	−1.0262	0.9802

**Table 5:** The affine matrix A of each Gaussian component of $T^{tar}$ with respect to $T^{src}$.

	New Task	$T^{\mathrm{tar}1}$	$T^{\mathrm{tar}2}$	$T^{\mathrm{tar}3}$
Affine Matrix				
$A_{1}$		$\left[\begin{array}{cc} \;-0.1194 & 0\\ 0 & 1.2280 \end{array}\right]$	$\left[\begin{array}{cc} -2.5144 & 0\\ 0 & 0.1692 \end{array}\right]$	$\left[\begin{array}{cc} 0.9828 & 0\\ 0 & -1.4648 \end{array}\right]$
$A_{2}$		$\left[\begin{array}{cc} \;2.0894 & 0\\ 0 & 0.4196 \end{array}\right]$	$\left[\begin{array}{cc} 2.0163 & 0\\ 0 & -0.7109 \end{array}\right]$	$\left[\begin{array}{cc} 1.7728 & 0\\ 0 & -0.5704 \end{array}\right]$
$A_{3}$		$\left[\begin{array}{cc} \;1.2050 & 0\\ 0 & 0.3530 \end{array}\right]$	$\left[\begin{array}{cc} -2.2734 & 0\\ 0 & 0.6326 \end{array}\right]$	$\left[\begin{array}{cc} 1.5997 & 0\\ 0 & -0.6386 \end{array}\right]$
$A_{4}$		$\left[\begin{array}{cc} \;3.7190 & 0\\ 0 & 0.6223 \end{array}\right]$	$\left[\begin{array}{cc} 6.3141 & 0\\ 0 & -0.2024 \end{array}\right]$	$\left[\begin{array}{cc} 1.3160 & 0\\ 0 & -1.7833 \end{array}\right]$

## Data Availability

Dataset available on request from the authors.

## Nomenclature

The following abbreviations are used in this manuscript:

Abbreviations	
GMM	Gaussian Mixture Model
GMR	Gaussian Mixture Regression
MKT	Motion Knowledge Transfer
LfD	Learning from Demonstration
Mathematical Symbol	
$\pi_{k}$	Weight of the *k*-th Gaussian component
$\mu_{k}$	Mean vector of the *k*-th Gaussian component
$\Sigma_{k}$	Covariance matrix of the *k*-th Gaussian component
*K*	Total number of Gaussian components
$\beta_{k}\left(t\right)$	Weight distribution of the *k*-th component at time *t*
$\kappa_{s,k}$	Sign function for covariance matrix adaptation
$A_{k}$	Affine transformation matrix
x(t)	Robot trajectory position at time *t*
$\dot{x}\left(t\right)$	Velocity of robot trajectory at time *t*
$\ddot{x}\left(t\right)$	Acceleration of robot trajectory at time *t*
*t*	Time variable
N	Gaussian distribution function
$R^{3}$	3D Cartesian space

## Appendix A

(A1) $$\begin{array}{c} t_{k}=\frac{\mu_{t,k+1}\Sigma_{t,k}-\mu_{t,k}\Sigma_{t,k+1}+B}{\Sigma_{t,k}-\Sigma_{t,k+1}}-\frac{\mu_{t,k}\Sigma_{t,k+1}-\mu_{t,k+1}\Sigma_{t,k}+B}{\Sigma_{t,k}-\Sigma_{t,k+1}} \end{array}$$

where

(A2) $$\begin{array}{c} B=\Sigma_{t,k}\Sigma_{t,k+1}\sqrt{\left(\frac{({\left(\mu_{t,k}\right)}^{2}-2\mu_{t,k}\mu_{t,k+1}+{\left(\mu_{t,k+1}\right)}^{2}-2\Sigma_{t,k}log(\frac{\pi_{k}\sqrt{\Sigma_{t,k+1}}}{\pi_{k+1}\sqrt{\Sigma_{t,k}}})+2\Sigma_{t,k+1}log(\frac{\pi_{k}\sqrt{\Sigma_{t,k+1}}}{\pi_{k+1}\sqrt{\Sigma_{t,k}}}))}{\Sigma_{t,k}\Sigma_{t,k+1}}\right)} \end{array}$$
